# Association between unpredictable work schedule changes and sleep disorders among wage workers: evidence from national Working Conditions Surveys in Korea and Europe

**DOI:** 10.1093/joccuh/uiag044

**Published:** 2026-07-31

**Authors:** Min Young Park, Jong-Su Kim, Joonho Ahn

**Affiliations:** Department of Occupational and Environmental Medicine, Seoul St Mary’s Hospital, College of Medicine, The Catholic University of Korea, Seoul, Republic of Korea; Department of Translational Medicine, Seoul National University College of Medicine, Seoul, Republic of Korea; Department of Occupational and Environmental Medicine, Kangbuk Samsung Hospital, Sungkyunkwan University School of Medicine, 29 Saemunan-ro, Jongno-gu, Seoul 03181, Republic of Korea

**Keywords:** personnel staffing and scheduling, sleep disorders, circadian rhythm, Europe, Republic of Korea, occupational exposure

## Abstract

**Objectives:**

Unpredictable work schedule changes have emerged as a distinct occupational health concern, yet their association with chronic sleep disorders remains understudied. Given the contrasting institutional contexts of the Republic of Korea and Europe, we hypothesized that the association would differ in magnitude between the 2 regions and examined this using a dose–response framework based on advance notice timing.

**Methods:**

This cross-sectional study analyzed data from the 2017 Korean Working Conditions Survey and the 2015 European Working Conditions Survey. Habitual work schedule unpredictability, reflecting structural instability in working time arrangements, was assessed by advance notice timing. Sleep disorders were assessed using the validated Minimal Insomnia Symptom Scale. Stratified and sensitivity analyses were performed.

**Results:**

Unpredictable schedule changes were associated with higher odds of sleep disorders in Korea (adjusted odds ratio [aOR] = 2.544; 95% CI, 2.195-2.948) and Europe (aOR = 1.787; 95% CI, 1.543-2.070), with a formally confirmed regional difference (*P* for interaction <.0001). Risk increased progressively as advance notice shortened, demonstrating a clear dose–response relationship. Among subgroups, a statistically significant interaction was identified for long working hours in Korea, with stronger associations observed among those without overtime.

**Conclusions:**

Habitual work schedule unpredictability is a distinct occupational risk factor for sleep disorders. The observed dose–response pattern suggests that greater advance notice may be associated with lower risk, highlighting schedule predictability as a potential occupational health priority warranting regulatory and organizational attention.

## Teaser

**Key points**
 

- **What is already known on this topic:** Irregular and long working hours, as well as shift work, are established risk factors for sleep disturbances and adverse health outcomes. While international organizations have identified unpredictable work schedules as an emerging occupational hazard, the specific relationship between schedule change predictability and sleep disorders has rarely been directly and systematically examined, particularly in a cross-national context.
- **What this study adds:** Using large, harmonized, nationally representative datasets from the Republic of Korea and Europe, this study demonstrates that unpredictable work schedule changes, defined as receiving notice on the same day or the day before, are independently associated with sleep disorder in both regions. A clear dose–response relationship was identified, whereby shorter advance notice periods corresponded to progressively greater risk of sleep problems, with the association being stronger in Korea than in Europe. Subgroup analyses identified long working hours as a statistically significant effect modifier in Korea, with stronger associations observed among workers without overtime.
- **How this study might affect research, practice, or policy:** These findings establish schedule predictability as a distinct and modifiable occupational risk factor for sleep disorders. Policymakers should consider mandating minimum advance notice requirements for schedule changes, particularly in sectors characterized by precarious employment. Future longitudinal and intervention studies are warranted to evaluate whether improving schedule predictability can meaningfully prevent sleep disorders and related health outcomes.

## 1. Introduction

In the modern labor market, working hours have evolved from traditional fixed patterns to multidimensional arrangements characterized by flexibility, irregularity, and variability.^1^^,^^2^ Among the various dimensions of working time, the predictability of work schedules, defined as the degree to which workers receive sufficient advance notice of changes to their scheduled hours, has emerged as a distinct and consequential feature of employment quality.^3^ International organizations such as the International Labour Organization and Eurofound have identified unpredictable work schedules as an emerging labor risk factor, drawing increasing policy attention.^4^^,^^5^ Low predictability in work schedules undermines the ability to plan daily activities, including family life, social interactions, self-care, and sleep, ultimately compromising health and overall quality of life.^6^^,^^7^

Although irregular and unpredictable work schedules have been implicated in a wide range of adverse health outcomes, including metabolic syndrome, musculoskeletal pain,^8^^,^^9^ cardiovascular disease,^10^ and mental health deterioration,^11^ most evidence derives from research on shift work or long working hours rather than from direct assessment of schedule unpredictability as a distinct exposure. The proposed mechanisms through which such schedule-related exposures operate, including circadian rhythm disruption, stress sensitization, and impaired cognitive and attentional function,^12^ suggest that unpredictable schedule changes may exert adverse health effects through similar pathways. Among the range of potential health consequences, sleep disorders warrant particular attention. Sleep is uniquely sensitive to schedule timing and predictability, and disordered sleep serves as both a direct consequence of circadian disruption and a proximal pathway to downstream chronic conditions such as cardiovascular disease and metabolic dysfunction.^12^^,^^13^ Furthermore, unlike the acute occupational accidents previously associated with schedule unpredictability by our group,^14^ disordered sleep represents a chronic pathogenic process with distinct long-term health implications. Despite the scale of the problem, systematic investigations into the relationship between schedule change predictability and sleep disorders remain scarce.

Several prior studies have examined the relationship between working time characteristics and sleep disturbances. Research in the US service sector further demonstrated that shorter advance notice of schedule changes is associated with poorer sleep quality,^15^ and a cross-sectional study using the Korean Working Conditions Survey (KWCS) and the European Working Conditions Survey (EWCS) found that work schedule changes were associated with sleep disturbance among health care professionals, without examining a dose–response gradient or extending beyond a single occupational group.^16^ These findings provide an important foundation, yet further evidence is needed. Studies extending beyond specific industries or occupational groups are required to establish generalizability across the broader workforce. Research capturing the gradient of advance notice timing, rather than treating schedule changes as a simple binary exposure, would allow for dose–response assessment. Investigations employing validated instruments for sleep disturbance are also warranted to ensure comparability across settings. Whether unpredictable schedule changes independently affect sleep disorders across the general working population, and whether the degree of advance notice matters in a dose–response manner with direct policy relevance, thus remain unexamined.

To address these gaps, this study examined the association between habitual unpredictable work schedule changes and chronic sleep disorders using harmonized nationally representative datasets from the Republic of Korea (KWCS) and Europe (EWCS),^17^ covering wage workers in general, rather than a single occupational group, and employing a dose–response framework based on advance notice timing. We hypothesized that shorter advance notice would correspond to progressively greater risk of sleep disorders, and that, given the contrasting institutional contexts between the 2 regions,^18^^,^^19^ the magnitude of this association would differ significantly, with a stronger effect expected in Korea.

## 2. Methods

### 2.1 Data source and study population

This cross-sectional study analyzed 2 nationally representative datasets, the 2017 KWCS and the 2015 EWCS. Both surveys were developed to comprehensively assess working environments and to support the formulation of labor and occupational health policies. Although conducted independently by Korea’s Occupational Safety and Health Research Institute and the European Foundation for the Improvement of Living and Working Conditions (Eurofound), the KWCS was intentionally modeled after the EWCS to allow meaningful international comparisons.^17^

In the fifth round of the KWCS, data were collected from over 50 000 economically active individuals aged 15 years or older across the Republic of Korea by using a multistage stratified random sampling method. The sixth EWCS, conducted in 2015, surveyed approximately 44 000 workers across 35 European countries. Despite differences in regional context and survey administration, the 2 datasets are comparable in terms of questionnaire structure, methodology, and key thematic domains, including working time arrangements, physical and psychosocial work environments, health outcomes, and job satisfaction. These structural and conceptual alignments provide a strong basis for cross-national comparisons. In this study, these harmonized instruments were leveraged to examine the relationship between unpredictable work schedules and adverse health outcomes by employing parallel analytical strategies for both datasets.

**Figure 1:**
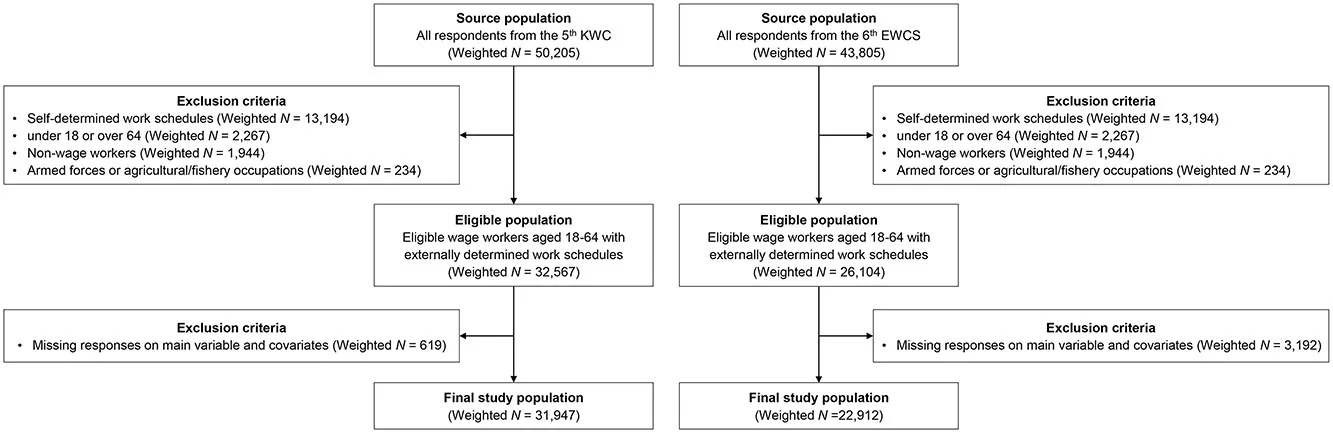
Schematic diagram of study participants from the fifth KWCS (2017) and the sixth EWCS (2015). Weighted N values were calculated using survey-provided sampling weights. EWCS, European Working Conditions Survey; KWCS, Korean Working Conditions Survey.

To enhance the validity of cross-national comparisons, the study population was defined based on harmonized inclusion and exclusion criteria across the fifth KWCS (2017) and sixth EWCS (2015) datasets (Figure 1). The analysis was restricted to wage workers aged between 18 and 64 years, whose work schedules were externally determined by their employers. Respondents who reported self-determined work schedules were excluded to ensure comparability of the exposure assessment. In both surveys, individuals outside the working-age population (<18 years or >64 years) and those not classified as wage earners (including employers, self-employed workers, and unpaid family workers) were excluded. Additionally, individuals employed in the armed forces or the agriculture/fishery sectors were excluded to improve occupational comparability, as their work schedules are inherently governed by strict hierarchical commands or uncontrollable environmental factors (eg, weather and seasonality) and are largely exempt from standard civilian labor regulations regarding working hours. Applying these criteria resulted in an eligible population of 32 567 respondents in the KWCS and 26 104 in the EWCS. Further exclusion of respondents for whom values on exposure or covariates were missing, yielded a final analytical sample of 31 947 for the KWCS and 22 532 for the EWCS.

### 2.2 Exposure and outcome variables

The primary exposure variable was the unpredictability of work schedule changes. Respondents were asked, “When regular changes to your work schedule occur, how far in advance are you informed?” They could indicate that no such changes ever occurred or that they were informed either on the same day, the day before, several days before, or several weeks before the change. To evaluate the dose–response relationship, the responses were categorized into 4 groups based on the timing of notification, allowing the assessment of a gradient effect. Furthermore, to identify those exposed to acutely unpredictable work schedules, a binary categorization was also employed as a primary approach. In this binary model, respondents who reported being informed on the same day or the day before a change were classified into the unpredictable-schedule group, to specifically capture the impact of sudden and immediate schedule disruptions. All other respondents were treated as the reference group. Recognizing that this binary cutoff is not an absolute threshold, we subsequently broadened the exposure definition to include respondents who received notice several days in advance in a sensitivity analysis, rigorously testing whether the results were sensitive to variations in how schedule unpredictability was operationalized.

The main outcome variable was sleep disorders, assessed using the Minimal Insomnia Symptom Scale (MISS),^20^ a validated brief screening instrument for sleep difficulties. The MISS comprises a 3-item battery evaluating difficulty falling asleep, waking up repeatedly during sleep, and waking up feeling exhausted over the previous 12 months. Each item was rated on a 5-point frequency scale and reverse-coded, yielding a total score ranging from 0 to 12, with a score of ≥6 used to classify individuals as having sleep disorder in accordance with established MISS criteria. This identical 3-item scale is employed in both surveys, ensuring full comparability of the outcome measure across datasets.

### 2.3 Other variables

**Figure 2:**
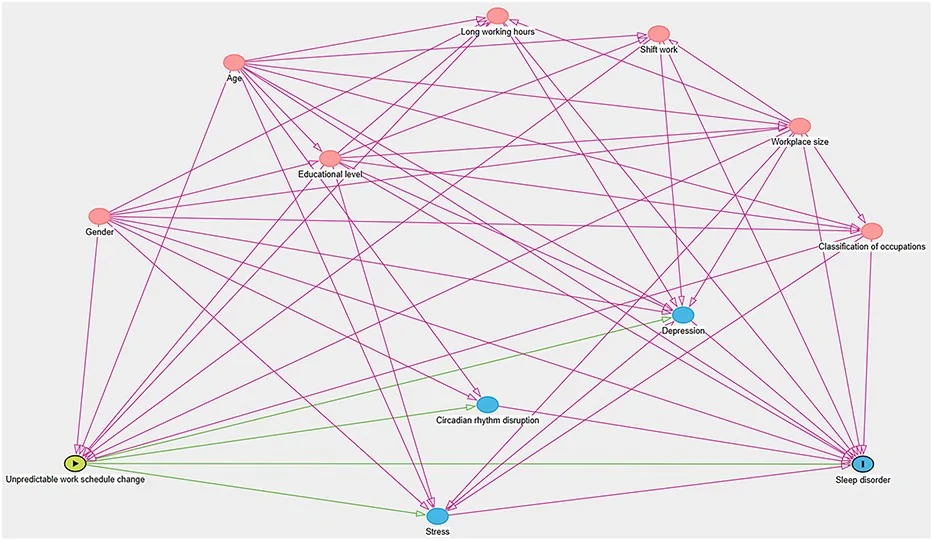
Directed acyclic graph (DAG) for the selection of adjustment variables. The main exposure variable, *unpredictable work schedule change*, is indicated by the node marked with a triangle (▶), and the outcome variable, *sleep disorder*, is represented by the node with an “I” symbol. Adjustment variables suggested by DAGitty (age, gender, educational level, workplace size, long working hours, shift work, and classification of occupations) are indicated in the graph; variables not selected for adjustment (depression, circadian rhythm disruption, and stress) are also shown. This figure was created using DAGitty (www.dagitty.net).

To account for potential confounding factors and strengthen the causal inference, a set of theoretically grounded covariates was identified based on a directed acyclic graph (DAG) approach (Figure 2). Rather than relying solely on statistical associations, which may lead to overadjustment or collider bias, variables were selected based on their role within the presumed causal structure linking exposure to the outcome. The adjustment set included age, sex, educational attainment, classification of occupation, long working hours, shift work, and workplace size. Age was treated as a continuous variable in the main adjustment models. For the purpose of stratified analyses, age was categorized into 2 groups (18-39 and 40-64 years) to capture potential differences across early-to-mid career stages and middle-aged physiological changes. Sex was categorized as male or female. Educational level was categorized as low education (high school or lower) or high education (college or higher). Classification of occupation was classified into 3 occupational groups—white-, pink-, and blue-collar work—based on the International Standard Classification of Occupations (ISCO-08) for the EWCS and the highly compatible Korean Standard Classification of Occupations (KSCO) for the KWCS. Specifically, white-collar work included managers, professionals, technicians, and clerical support workers (ISCO-08 codes 1-4; KSCO codes 1-3). Pink-collar work comprised service and sales workers (ISCO-08 code 5; KSCO codes 4-5). Blue-collar work included craft and related trades workers, plant and machine operators, and elementary occupations (ISCO-08 codes 7-9; KSCO codes 7-9). Long working hours were defined as working more than 52 hours per week, and shift work was identified by responses indicating regular engagement in shift-based schedules. Workplace size was categorized based on the number of employees, with small and medium-sized enterprises defined as having fewer than 250 employees, and large enterprises defined as having 250 or more employees. All variables in the adjustment set were selected to minimize confounding based on causal assumptions, rather than empirical correlations alone.

In addition to the covariates, mental health status was conceptualized as a potential mediator in the DAG and was therefore used exclusively to explore potential effect differences to avoid overadjustment bias. Depressive symptoms were assessed using the WHO-5 Well-being Index, which is a validated screening tool for emotional well-being. The index consists of 5 positively worded items, each scored from 0 (“At no time”) to 5 (“All of the time”), with the total raw score ranging from 0 to 25. This raw score is multiplied by 4 to yield a percentage score ranging from 0 to 100. Following the recommendations of the World Health Organization, a score of <50 was used to define poor mental well-being, indicating possible depression. This dichotomized variable was included in the stratified analyses to assess potential effect modification.

### 2.4 Statistical analysis

Weighted binomial logistic regression models were used to reflect the sampling structure of the survey data. The results are presented as odds ratios (ORs) with 95% CIs. As a primary analytical step to establish a gradient effect, we categorized respondents into 4 groups based on their reported timing of notification to evaluate a dose–response association. Those who reported no schedule changes or who were informed several weeks in advance served as the reference group. Comparisons were made with 3 other groups: those who were notified several days before, on the day before, and on the same day. To statistically evaluate this dose–response relationship, a *P* for trend was calculated by treating the ordinal categories of notice timing as a continuous variable in the models.

Following the dose–response analysis, a binary categorization (same day or day before vs others) was applied to the main and stratified analyses to evaluate the effects of schedule changes. Furthermore, to formally evaluate the regional differences in the magnitude of the association, a formal interaction test was conducted using a combined dataset, which included a region-by-exposure interaction term. To explore the potential differences in associations across population subgroups using this binary exposure, stratified models were constructed according to sex, age group (18-39 vs 40-64 years), classification of occupation, workplace size, shiftwork status, weekly working hours, educational level, and depressive symptoms. To statistically evaluate the differences between these subgroups, a *P* for interaction was calculated for each stratified model. Furthermore, to aid in the clinical and epidemiological interpretation of the findings, the prevalence of sleep disorders was examined for subgroups that demonstrated statistically significant interactions. Each model was adjusted for a consistent set of covariates derived from a DAG, excluding stratifying variables within each model.

To assess the robustness of the findings, 3 sensitivity analyses were performed. First, a quasi-binomial model was applied to account for potential overdispersion in the survey data. Second, the regression models were re-estimated without applying the survey weights. Third, the definition of exposure was broadened to include respondents who received notice several days in advance, testing whether the results were sensitive to variations in how schedule unpredictability was operationalized. All statistical analyses were performed using R software version 4.4.3 (64-bit) (R Foundation for Statistical Computing, Vienna, Austria).

This study was exempted from review by the Institutional Review Board of Kangbuk Samsung Hospital (IRB No. KBSMC 2025-08-072), as it used publicly available, de-identified secondary data.

## 3. Results

The characteristics of the participants stratified by the presence of unpredictable work schedule changes in both the KWCS and EWCS are shown in Table 1. The average (±SD) age was highly comparable between the reference and unpredictable-schedule groups in both the KWCS (41.1 ± 11.3 vs 41.2 ± 11.7 years) and the EWCS (40.3 ± 11.7 vs 40.3 ± 11.7 years), showing balanced distributions across both the younger (18-39) and middle-aged (40-64) categories. In both surveys, the proportion of male workers was higher in the unpredictable-schedule group than in the reference group. In the KWCS, the proportion of workers with higher educational attainment was lower among those with unpredictable schedules (59.8%) than among those without (64.4%), while in the EWCS, this difference was much smaller (26.6% vs 28.8%). White-collar work accounted for a smaller proportion of the unpredictable-schedule group, whereas pink- and blue-collar occupations were more prevalent among those with unpredictable schedules in both datasets. Regarding workplace size, workers in small- and medium-sized enterprises comprised the vast majority across both groups in the KWCS (89.7% and 91.8%), whereas in the EWCS, large enterprises accounted for approximately one-third of the workforce in both groups (31.4% and 34.3%). The unpredictable-schedule group showed a higher prevalence of long working hours, shift work, depressive symptoms, and sleep problems in both surveys, with more pronounced differences observed in the EWCS.

**Table 1:** Characteristics of study participants by unpredictable work schedule changes in KWCS and EWCS.

Characteristics	KWCS			EWCS		
	Unpredictable work schedule change			Unpredictable work schedule change		
	No	Yes	*P* value ^a^	No	Yes	*P* value ^a^
**Gender**						
**Male**	15 678 ± 160 (56.3 ± 0.4%)	2554 ± 72 (62.2 ± 1.1%)	<.0001	10 380 ± 159 (51.7 ± 0.6%)	1679 ± 68 (59.2 ± 1.5%)	<.0001
**Female**	12 161 ± 122 (43.7 ± 0.4%)	1554 ± 53 (37.8 ± 1.1%)	–	9698 ± 152 (48.3 ± 0.6%)	1155 ± 53 (40.8 ± 1.5%)	–
**Age** ^**b**^	41.1 ± 11.3	41.2 ± 11.7	.7006	40.3 ± 11.7	40.3 ± 11.7	.9966
**Age group**						
**18-39**	12 979 ± 154 (46.6 ± 0.4%)	1879 ± 66 (45.7 ± 1.1%)	.4625	9573 ± 159 (47.7 ± 0.6%)	1336 ± 58 (47.1 ± 1.5%)	.7387
**40-64**	14 859 ± 130 (53.4 ± 0.4%)	2229 ± 60 (54.3 ± 1.1%)	–	10 505 ± 152 (52.3 ± 0.6%)	1498 ± 64 (52.9 ± 1.5%)	–
**Educational level** ^**c**^						
**Low**	9915 ± 116 (35.6 ± 0.4%)	1650 ± 52 (40.2 ± 1.1%)	<.0001	14 297 ± 182 (71.2 ± 0.5%)	2079 ± 75 (73.4 ± 1.3%)	.1357
**High**	17 923 ± 161 (64.4 ± 0.4%)	2459 ± 72 (59.8 ± 1.1%)	–	5781 ± 117 (28.8 ± 0.5%)	755 ± 42 (26.6 ± 1.3%)	–
**Classification of occupations**						
**White-collar occupation**	14 718 ± 155 (52.9 ± 0.4%)	1768 ± 63 (43.0 ± 1.1%)	<.0001	8248 ± 154 (41.8 ± 0.6%)	836 ± 51 (30.1 ± 1.5%)	<.0001
**Pink-collar occupation**	5323 ± 83 (19.1 ± 0.3%)	997 ± 42 (24.3 ± 0.9%)	–	4328 ± 96 (21.9 ± 0.5%)	789 ± 42 (28.4 ± 1.3%)	–
**Blue-collar occupation**	7797 ± 113 (28.0 ± 0.4%)	1343 ± 49 (32.7 ± 1.0%)	–	7175 ± 131 (36.3 ± 0.6%)	1156 ± 54 (41.6 ± 1.5%)	–
**Workplace size**						
**Small & medium**	24 974 ± 158 (89.7 ± 0.3%)	3770 ± 84 (91.8 ± 0.6%)	.0041	13 766 ± 173 (68.6 ± 0.6%)	1862 ± 66 (65.7 ± 1.5%)	.0717
**Large**	2864 ± 77 (10.3 ± 0.3%)	339 ± 26 (8.2 ± 0.6%)	–	6312 ± 132 (31.4 ± 0.6%)	972 ± 55 (34.3 ± 1.5%)	–
**Long working hours**						
**No**	24 805 ± 160 (89.1 ± 0.3%)	3490 ± 81 (84.9 ± 0.8%)	<.0001	18 836 ± 195 (93.8 ± 0.3%)	2579 ± 83 (91.0 ± 0.7%)	.0001
**Yes**	3034 ± 74 (10.9 ± 0.3%)	619 ± 34 (15.1 ± 0.8%)	–	1242 ± 61 (6.2 ± 0.3%)	255 ± 22 (9.0 ± 0.7%)	–
**Shift work**						
**No**	24 915 ± 160 (89.5 ± 0.3%)	3415 ± 80 (83.1 ± 0.8%)	<.0001	14 668 ± 182 (73.1 ± 0.5%)	1725 ± 68 (60.9 ± 1.5%)	<.0001
**Yes**	2924 ± 73 (10.5 ± 0.3%)	694 ± 37 (16.9 ± 0.8%)	–	5410 ± 116 (26.9 ± 0.5%)	1109 ± 53 (39.1 ± 1.5%)	–
**Depression**						
**No**	20 429 ± 156 (73.4 ± 0.4%)	3150 ± 78 (76.7 ± 0.9%)	.0014	16 963 ± 190 (84.5 ± 0.4%)	2128 ± 73 (75.1 ± 1.4%)	<.0001
**Yes**	7409 ± 113 (26.6 ± 0.4%)	959 ± 42 (23.3 ± 0.9%)	–	3116 ± 91 (15.5 ± 0.4%)	707 ± 45 (24.9 ± 1.4%)	–
**Sleep disorder**						
**No**	26 349 ± 160 (94.6 ± 0.2%)	3584 ± 83 (87.2 ± 0.7%)	<.0001	15 944 ± 187 (79.4 ± 0.5%)	1925 ± 69 (67.9 ± 1.5%)	<.0001
**Yes**	1490 ± 52 (5.4 ± 0.2%)	525 ± 31 (12.8 ± 0.7%)	–	4134 ± 103 (20.6 ± 0.5%)	909 ± 51 (32.1 ± 1.5%)	–

Abbreviations: EWCS, European Working Conditions Survey; KWCS, Korean Working Conditions Survey.

Values are presented as weighted estimates with standard errors: weighted number ± SE (weighted percentage ± SE), except for age.

^a^ *P* values were calculated using the survey-weighted chi-squared test for categorical variables and the survey-weighted *t* test for age.

^b^Continuous age is presented as survey-weighted mean ± SD.

^c^Educational level was grouped as low education (≤high school) and high education (≥college).

**Figure 3:**
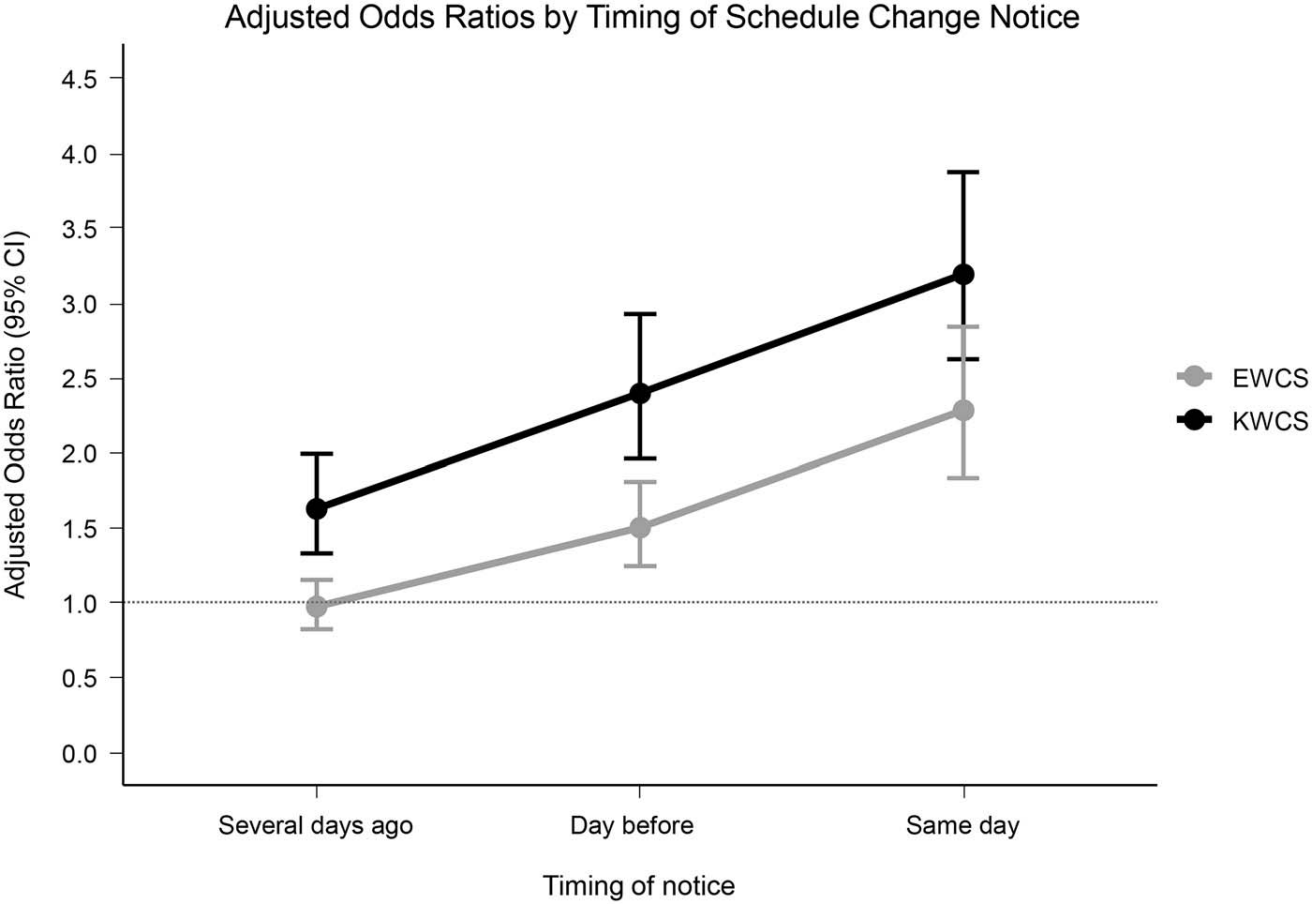
Risk of sleep disorder by timing of schedule change notice. Adjusted odds ratios (aORs) were estimated after controlling for age, gender, educational level, workplace size, long working hours, classification of occupation, and shift work. Dots represent point estimates of ORs, and vertical lines indicate 95% CIs. The reference group comprises those who responded either “No change occurred” or “Notified several weeks in advance.” EWCS, European Working Conditions Survey; KWCS, Korean Working Conditions Survey.

Figure 3 shows the dose–response association between the timing of the schedule change notice and the risk of sleep disorders. Using those who reported no schedule changes or received notice several weeks in advance as the reference group, we observed a clear gradient in the adjusted ORs across both the KWCS and the EWCS. In both surveys, the risk of sleep disorders progressively increased as the timing of the notice decreased (*P* for trend <.0001 in both surveys). In the KWCS, the adjusted ORs increased from approximately 1.6 for those notified several days in advance to 2.4 for those notified the day before, and further to >3.2 for those notified on the same day. A similar increasing pattern was observed in the EWCS, although the magnitude of the association was smaller: the OR increased from 1.1 to 1.5 and reached approximately 2.2 for same-day notice. This trend suggests a consistent dose–response relationship, whereby a short period of advance notice is associated with an increased likelihood of sleep disorders. Sensitivity analyses corresponding to Figure 3 are presented in Table S1, and the associations remained consistent across alternative specifications, with all models indicating highly significant dose–response gradients (*P* for trend <.0001), further supporting the robustness of the dose–response findings.

Unpredictable work schedule changes were significantly associated with higher odds of sleep disorder in both the KWCS and EWCS (Table 2). The association was stronger in the KWCS (adjusted OR [aOR] = 2.544; 95% CI, 2.195-2.948) than in the EWCS (aOR = 1.787; 95% CI, 1.543-2.070), although both were statistically robust. Sensitivity analyses using alternative modeling strategies and exposure definitions are shown in Table S2. Across all approaches, the associations remained consistently significant and in the expected direction, supporting the robustness of the main findings. A region-by-exposure interaction test showed that this significant regional difference was robustly maintained across both the main and all sensitivity analyses (all *P* for interaction <.0001).

**Table 2:** Comparison of crude and adjusted odds ratios (ORs) for sleep disorders in KWCS and EWCS.

Model	KWCS	EWCS
**Crude OR (95% CI)**	2.592 (2.242-2.997)	1.810 (1.566-2.092)
**Adjusted OR (95% CI)**	2.544 (2.195-2.948)	1.787 (1.543-2.070)

Abbreviations: EWCS, European Working Conditions Survey; KWCS, Korean Working Conditions Survey.

Adjusted for age, gender, education level, workplace size, long working hours, classification of occupation, and shift work.

**Figure 4:**
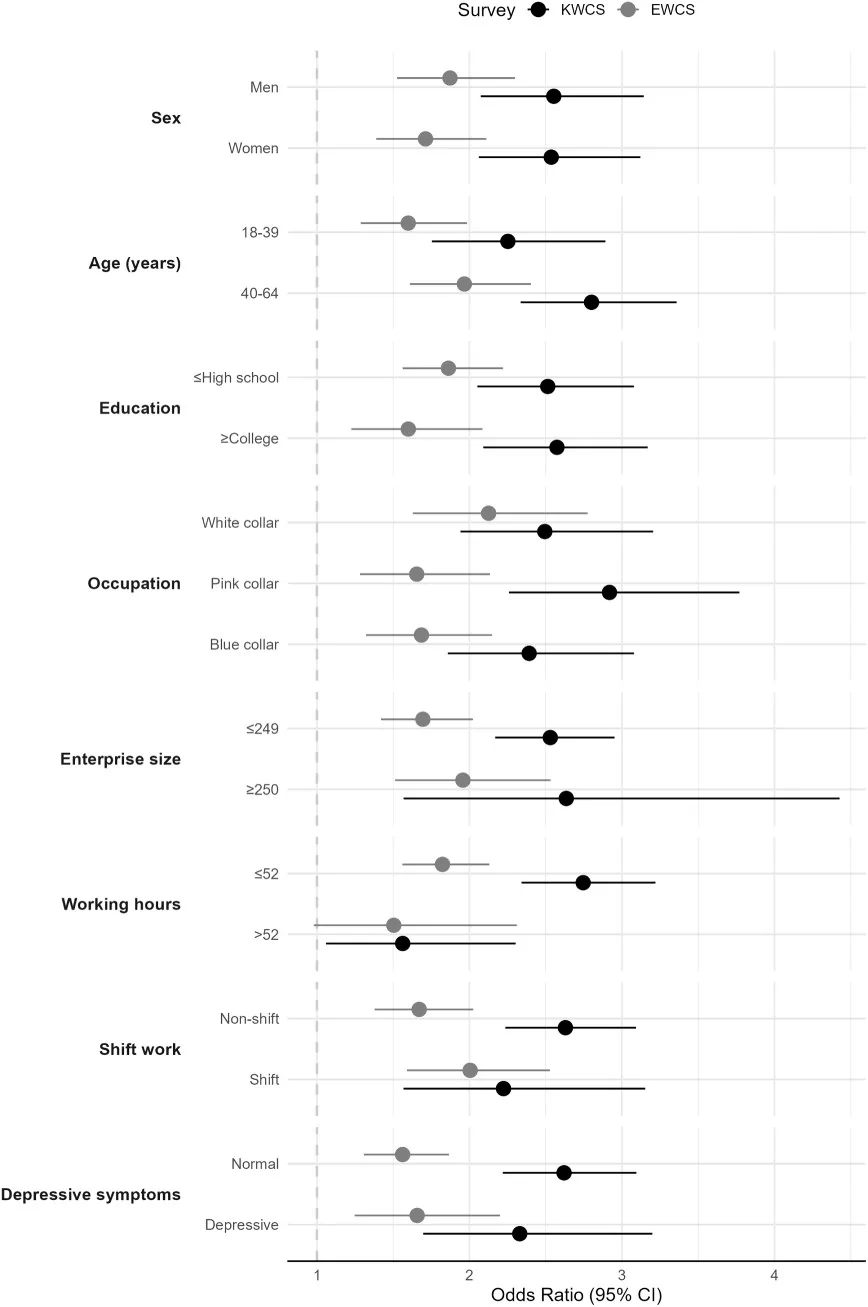
Stratified analysis of the association between unpredictable work schedule changes and sleep disorder. Adjusted odds ratios (aORs) were calculated after adjusting for age, gender, educational level, workplace size, long working hours, classification of occupation, and shift work. In each stratified analysis, the stratification variable was excluded from the adjustment. Dots represent point estimates of ORs, and vertical lines indicate 95% CIs.

Stratified analyses revealed notable variations in the magnitude of the association between unpredictable work schedule changes and sleep disorders across the subgroups (Figure 4 and Table S2). In the KWCS, long working hours showed a robust and statistically significant interaction across the main and multiple sensitivity analyses (*P* for interaction = .0125 in the main analysis, =.0132 in the first sensitivity analysis, and =.0051 in the second sensitivity analysis). Specifically, the association between unpredictable work schedule changes and sleep disorders was stronger among those who did not work long hours (OR = 2.746; 95% CI, 2.342-3.220) than among those who worked long hours (OR = 1.562; 95% CI, 1.060-2.303), a finding contextualized by the baseline prevalence of sleep disorders, which was 7.9 ± 0.6% among those working long hours compared with 6.1 ± 0.2% among those without. In contrast, no significant interaction for long working hours was observed in the EWCS (*P* for interaction = .3503). For other stratifying variables, interaction effects were either completely nonsignificant or showed only sporadic significance in isolated sensitivity analyses, lacking robust and consistent patterns across alternative specifications.

## 4. Discussion

In this study, we found that unpredictable work schedule changes were associated with an increased risk of sleep disorders in both Korea and Europe, with the association significantly stronger in Korea. The risk of sleep disorders increased as the notice period for schedule changes shortened, confirming a dose–response relationship. Among the stratifying variables examined, long working hours was the only subgroup factor showing a statistically significant interaction, an effect observed specifically in Korea.

Work schedule unpredictability is associated with sleep disorders. While our prior work demonstrated that schedule unpredictability elevates acute accident risk by compromising immediate cognitive readiness through circadian disruption and fatigue,^14^ the present findings suggest a more protracted pathogenic process—one driven by chronic biological rhythm dysregulation and cumulative psychosocial strain. One plausible explanation is that sudden changes in work schedules make it difficult for workers to pre-adjust their sleep–wake cycles. Even without the repetitive disruption characteristics seen in shift work, irregularities can create a mismatch between endogenous circadian rhythms and external demands, thereby impairing sleep onset and maintenance.^13^^,^^21^ Furthermore, unpredictability itself might act as a psychosocial stressor, and the uncertainty of whether one will be required to work on short notice can induce anticipatory stress, elevate arousal levels, and interfere with sleep.^7^^,^^22^ In addition, schedule unpredictability can disrupt daily routines such as meal times and physical activity, contributing to poor sleep hygiene and confusion regarding lifestyle-related factors.^12^ Sudden changes in family and social responsibilities, such as caregiving duties, social commitments, and household chores, may compound these effects, and their accumulation can heighten psychological strain, ultimately leading to sleep disturbances.^6^^,^^7^

This study identified a dose–response relationship, showing that the shorter the advance notice period for schedule changes, the greater the risk of sleep disorders. This gradient indicates that not only the occurrence of schedule unpredictability but also the extent of advance notice play a critical role in workers’ sleep health. From an occupational standpoint, the dose–response pattern suggests that the degree of schedule unpredictability, not merely its occurrence, is what drives harm, raising the possibility that incremental improvements in advance notice could be associated with corresponding reductions in sleep disorder risk. Notably, workers exposed to same-day notice faced more than 3 times the odds of sleep disorders in Korea, underscoring the acute burden imposed by the most extreme form of schedule unpredictability. These findings are consistent with prior evidence from the US service sector linking shorter advance notice to poorer sleep quality,^15^ and extend that evidence by demonstrating a systematic gradient across multiple notice timing categories in 2 nationally representative populations.

The stronger association between unpredictable work schedule changes and sleep disorders observed in Korea may reflect contextual differences in labor characteristics and institutional context between the 2 regions. In Europe, regulatory frameworks across many countries provide workers with concrete protections against sudden schedule changes, including minimum advance notice requirements, rights to refuse last-minute changes, compensatory rest provision, and work-life balance entitlements.^18^^,^^23^^,^^24^ These mechanisms may attenuate the psychological and physiological burden of schedule unpredictability even when changes do occur. It should be noted, however, that Europe encompasses a diverse range of countries with substantially varying labor regulations and cultural norms, and the observed association in the EWCS likely reflects this heterogeneity. In contrast, Korea is characterized by a persistent culture of long working hours, hierarchical workplace power dynamics that limit workers’ ability to refuse sudden schedule demands, and a relatively low uptake of flexible work arrangements.^19^^,^^25^ Furthermore, the high prevalence of precarious employment in Korea^26^ further constrains workers’ capacity to negotiate or resist sudden schedule changes, while the large share of employment in the service sector,^27^ where rapidly changing schedules are often intrinsic to job demands, compounds this vulnerability.

In the KWCS, the association between unpredictable schedule changes and sleep disorders was stronger among workers without long working hours than among those working overtime. The higher baseline prevalence of sleep disorders among workers with long working hours (7.9% vs 6.1% in the KWCS) may partly explain this weaker association, as chronic time pressure and accumulated fatigue in the long working hours group may independently elevate sleep disorder risk, potentially limiting the additional impact attributable to schedule unpredictability alone.^28^^,^^29^ Other factors, such as overlapping risk factors or residual confounding, may also contribute, and this explanation should therefore be regarded as one plausible interpretation rather than a definitive one. Given the already elevated baseline risk of sleep disorders in this group, however, this pattern cannot be interpreted as justifying sudden schedule changes for workers with long working hours.

For other stratifying variables, including classification of occupation, shift work status, and depressive symptoms, interaction effects lacked robust and consistent patterns and are therefore interpreted with caution. Non–shift workers showed numerically larger effect sizes than shift workers in both datasets, although this did not reach statistical significance in interaction tests. For non–shift workers, unpredictable schedule changes may manifest as sudden overtime requests or unexpected shift extensions. Unlike the chronic circadian volatility of shift work, these might represent acute perturbations to an otherwise stable routine that warrant further investigation in future studies.

This study had several limitations. First, its cross-sectional design makes definite causal inferences difficult, and the reliance on self-reported measures for sleep disorders may have led to reporting bias. Second, sleep disorders were assessed using a simplified 3-item questionnaire without a clinical diagnosis or objective monitoring, which does not fully correspond to clinically defined insomnia. In addition, unpredictable schedules were operationalized by the timing of notification, whereas real-world workplaces might involve more complex dimensions, such as notification frequency, the presence of recurring patterns, sudden cancellations, and shift extension. Third, although the KWCS and EWCS are nationally representative, residual differences in the cultural context, survey administration, and labor regulations could have contributed to the observed discrepancies. Moreover, cross-national differences between Korea and Europe might reflect not only institutional and cultural contexts but also variations in response styles or stigma related to reporting sleep problems. Fourth, the KWCS (2017) and EWCS (2015) were conducted 2 years apart; although we selected the most contemporaneous rounds available, labor market or regulatory changes during this period may have affected comparability, and future studies using simultaneously collected data would further strengthen cross-national comparisons. Despite these limitations, this study draws on 2 large, nationally representative datasets with harmonized structures, enabling robust cross-national comparisons. The application of a DAG-based approach and examination of subgroup differences, dose–response patterns, and sensitivity analyses offers a comprehensive view of how schedule unpredictability influences sleep health.

These findings carry direct implications for occupational health practice and policy. Schedule predictability should be recognized as a distinct and modifiable occupational risk factor, and employers should be encouraged to provide workers with maximum feasible advance notice of schedule changes. At the regulatory level, policymakers should consider establishing minimum advance notice requirements for schedule changes, particularly in sectors characterized by precarious employment and high schedule volatility. Future longitudinal and intervention studies are warranted to evaluate whether improving schedule predictability can meaningfully reduce the burden of sleep disorders among workers.

## 5. Conclusions

This study, using harmonized data from Korea and Europe, showed that unpredictable work schedule changes were associated with a dose-dependent increase in sleep disorder risk among wage workers, with a significantly stronger association in Korea. Long working hours emerged as a significant effect modifier in Korea, with stronger associations observed among workers without overtime. Although the cross-sectional design and self-reported measures limit causal inference, the robustness of the findings across multiple sensitivity analyses supports the interpretation of schedule unpredictability as a distinct occupational risk factor for sleep disorders. These results highlight the need for regulatory and organizational measures to secure adequate advance notice of schedule changes and for future longitudinal and intervention studies to evaluate whether improving schedule predictability can prevent sleep disorders.

## Supplementary Material

Final_Supplementary_Materials_260730_uiag044

## Data Availability

The data underlying this article are available in the public domain. The datasets were derived from publicly accessible sources: the Korean Working Conditions Survey (KWCS), available from the Occupational Safety and Health Research Institute (OSHIRI; https://oshri.kosha.or.kr/eoshri/resources/KWCSDownload.do), and the European Working Conditions Survey (EWCS), available from Eurofound (https://www.eurofound.europa.eu/en/surveys-and-data/surveys/european-working-conditions-survey/ewcs-2015).
