# Supplementary material for: Association between unpredictable work schedule changes and sleep disorders among wage workers: evidence from national Working Conditions Surveys in Korea and Europe
Source: J Occup Health. 2026 Jul 31;68(1):uiag044. doi: 10.1093/joccuh/uiag044 (PMC13526365; doi:10.1093/joccuh/uiag044)
Supplement: Supplementary file 1 [file final_supplementary_materials_260730_uiag044.docx]

**Supplementary Information**

**Association between unpredictable work schedule changes and sleep disorders among wage workers: Evidence from national working conditions surveys in Korea and Europe**

Min Young Park^a^, Jong-Su Kim^b^ and Joonho Ahn^c,*^

^a^ Department of Occupational and Environmental Medicine, Seoul St. Mary’s Hospital, College

of Medicine, The Catholic University of Korea, Seoul, Republic of Korea

^b^ Department of Translational Medicine, Seoul National University College of Medicine, Seoul, Republic of Korea

^c^ Department of Occupational and Environmental Medicine, Kangbuk Samsung Hospital, Sungkyunkwan University School of Medicine, Seoul, Republic of Korea

***Corresponding author. Joonho Ahn**

Department of Occupational and Environmental Medicine, Kangbuk Samsung Hospital, Sungkyunkwan University School of Medicine, 29 Saemunan-ro, Jongno-gu, Seoul 03181, Korea. Email: [drcox@naver.com](mailto:drcox@naver.com)

**Table of Contents**

**Supplementary Tables**

Supplementary Table S1. Sensitivity analyses on the association between unpredictable work schedule changes and sleep disorder.................................................................................................................3

Supplementary Table S2. Sensitivity analyses of the risk of sleep disorder according to the timing of schedule change notification..............................................................................................................5

**Supplementary** Table S1. Sensitivity analyses of the risk of sleep disorder according to the timing of schedule change notification

| Timing of Schedule Change Notification | First Sensitivity Analysis | | Second Sensitivity Analysis | |
| --- | --- | --- | --- | --- |
|  | KWCS | EWCS | KWCS | EWCS |
| Several weeks in advance | 1.623(1.325-1.986) | 0.968(0.819-1.145) | 1.659(1.411-1.951) | 1.034(0.932-1.147) |
| Several days in advance | 2.388(1.955-2.918) | 1.496(1.241-1.803) | 2.200(1.883-2.571) | 1.356(1.209-1.520) |
| Day before or same-day notification | 3.184(2.619-3.870) | 2.275(1.826-2.834) | 4.299(3.703-4.989) | 1.862(1.639-2.116) |

***Notes*.** The presented odds ratios (ORs) are adjusted for age, gender, education level, workplace size, long working hours, classification of occupation and shift work. The reference group comprises those who responded either "No change occurred" or "Notified several weeks in advance". The first sensitivity analysis applied a quasi-binomial logistic regression model instead of a binomial model. The second sensitivity analysis used the same model but without applying survey weights (unweighted analysis).

KWCS = Korean Working Conditions Survey, EWCS = European Working Conditions Survey, OR = odds ratio, CI = confidence interval.

Supplementary Table S2. Sensitivity analyses on the association between unpredictable work schedule changes and sleep disorder

|  | First Sensitivity Analysis | | Second Sensitivity Analysis | | Third Sensitivity Analysis | |
| --- | --- | --- | --- | --- | --- | --- |
|  | KWCS | EWCS | KWCS | EWCS | KWCS | EWCS |
| Overall |  |  |  |  |  |  |
| Crude OR | 2.592(2.242-2.997) | 1.810(1.566-2.092) | 2.850(2.548-3.188) | 1.549(1.421-1.688) | 2.319(2.037-2.640) | 1.392(2.037-1.565) |
| Adjusted OR | 2.544(2.195-2.948) | 1.787(1.543-2.070) | 2.810(2.509-3.148) | 1.542(1.412-1.684) | 2.265(1.980-2.591) | 1.344(1.980-1.517) |
| Stratified results |  |  |  |  |  |  |
| Gender |  |  |  |  |  |  |
| Male | 2.553(2.074-3.143) | 1.873(1.525-2.299) | 2.346(1.981-2.780) | 1.520(1.338-1.728) | 2.214(1.829-2.682) | 1.398(1.829-1.667) |
| Female | 2.537(2.061-3.121) | 1.712(1.389-2.111) | 3.286(2.820-3.830) | 1.585(1.403-1.791) | 2.333(1.937-2.811) | 1.318(1.937-1.556) |
| Age group |  |  |  |  |  |  |
| 18-39 | 2.252(1.754-2.892) | 1.599(1.287-1.985) | 2.407(1.987-2.917) | 1.515(1.327-1.729) | 1.862(1.485-2.335) | 1.339(1.485-1.604) |
| 40-64 | 2.801(2.336-3.359) | 1.967(1.61-2.403) | 3.09(2.683-3.559) | 1.569(1.394-1.767) | 2.623(2.222-3.097) | 1.348(2.222-1.587) |
| Educational level |  |  |  |  |  |  |
| Low | 2.514(2.052-3.079) | 1.862(1.562-2.220) | 2.893(2.466-3.394) | 1.575(1.417-1.750) | 2.141(1.774-2.584) | 1.375(1.774-1.588) |
| High | 2.574(2.091-3.169) | 1.599(1.226-2.086) | 2.755(2.343-3.239) | 1.466(1.247-1.723) | 2.355(1.955-2.837) | 1.268(1.955-1.585) |
| Classification of occupations |  |  |  |  |  |  |
| White collar | 2.494(1.941-3.205) | 2.126(1.628-2.775) | 2.704(2.223-3.29) | 1.726(1.478-2.017) | 2.470(1.993-3.060) | 1.576(1.993-1.946) |
| Pink collar | 2.918(2.259-3.770) | 1.654(1.282-2.135) | 3.171(2.608-3.855) | 1.668(1.425-1.952) | 2.270(1.787-2.883) | 1.155(1.787-1.430) |
| Blue collar | 2.392(1.858-3.079) | 1.685(1.321-2.150) | 2.622(2.145-3.205) | 1.322(1.141-1.532) | 1.976(1.569-2.487) | 1.341(1.569-1.641) |
| Workplace size |  |  |  |  |  |  |
| Small and medium | 2.530(2.169-2.952) | 1.695(1.420-2.023) | 2.900(2.578-3.263) | 1.455(1.305-1.622) | 2.264(1.965-2.608) | 1.314(1.965-1.522) |
| Large | 2.635(1.569-4.427) | 1.957(1.513-2.532) | 1.898(1.222-2.949) | 1.721(1.479-2.003) | 2.233(1.421-3.509) | 1.392(1.421-1.716) |
| Long working hours |  |  |  |  |  |  |
| No | 2.746(2.342-3.220) | 1.823(1.561-2.130) | 3.002(2.657-3.391) | 1.537(1.400-1.688) | 2.362(2.043-2.730) | 1.333(2.043-1.514) |
| Yes | 1.562(1.060-2.303) | 1.504(0.979-2.311) | 1.828(1.333-2.505) | 1.580(1.205-2.073) | 1.711(1.208-2.423) | 1.447(1.208-2.192) |
| Shift work |  |  |  |  |  |  |
| No | 2.630(2.236-3.093) | 1.671(1.378-2.025) | 2.958(2.612-3.351) | 1.493(1.331-1.676) | 2.400(2.076-2.774) | 1.369(2.076-1.600) |
| Yes | 2.224(1.568-3.153) | 2.005(1.590-2.527) | 2.263(1.725-2.969) | 1.633(1.423-1.874) | 1.717(1.242-2.374) | 1.340(1.242-1.627) |
| Depression |  |  |  |  |  |  |
| No | 2.620(2.219-3.095) | 1.562(1.307-1.866) | 2.948(2.591-3.356) | 1.438(1.288-1.605) | 2.620(2.219-3.095) | 1.562(2.219-1.866) |
| Yes | 2.330(1.697-3.200) | 1.657(1.247-2.202) | 2.380(1.874-3.023) | 1.396(1.175-1.657) | 2.330(1.697-3.200) | 1.657(1.697-2.202) |

*Notes*. Except for the crude ORs, all presented odds ratios (ORs) were adjusted for age, gender, education level, workplace size, long working hours, classification of occupation and shift work. All values are presented as ORs with 95% confidence intervals (CIs).

The first sensitivity analysis applied a quasi-binomial logistic regression model instead of a binomial logistic regression model.

The second sensitivity analysis used the same model specification but without applying survey weights (unweighted analysis).

The third sensitivity analysis expanded the definition of the main exposure, including those who received schedule change notification several days in advance as part of the exposed group.

KWCS = Korean Working Conditions Survey, EWCS = European Working Conditions Survey, OR = odds ratio, CI = confidence interval.
